# Case report: Congenital mitral and tricuspid valve insufficiency in a patient with Axenfeld-Rieger syndrome

**DOI:** 10.3389/fcvm.2022.977432

**Published:** 2022-09-23

**Authors:** Jingwei Feng, Yingjiao Wang, Shiyu Cheng, Zishuo Liu, Ling Lan, Qi Miao, Chaoji Zhang

**Affiliations:** ^1^Department of Surgery, Peking Union Medical College Hospital, Peking Union Medical College, Chinese Academy of Medical Sciences, Beijing, China; ^2^Department of Ophthalmology, Peking Union Medical College Hospital, Peking Union Medical College, Chinese Academy of Medical Sciences, Beijing, China; ^3^Department of Ultrasound, Beijing Hospital, National Center of Gerontology, Institute of Geriatric Medicine, Chinese Academy of Medical Sciences, Beijing, China; ^4^Department of Anesthesiology, Peking Union Medical College Hospital, Peking Union Medical College, Chinese Academy of Medical Sciences, Beijing, China; ^5^Department of Cardiac Surgery, Peking Union Medical College Hospital, Peking Union Medical College, Chinese Academy of Medical Sciences, Beijing, China

**Keywords:** Axenfeld-Rieger syndrome (ARS), mitral valve insufficiency, tricuspid valve insufficiency, glaucoma, cardiac malformations

## Abstract

Axenfeld-Rieger syndrome (ARS) is an autosomal dominant disorder that is primarily due to disruption of the development of neural crest cells. The onset of associated symptoms in both eyes accompanied by extraocular developmental defects is referred to as ARS. Cardiac defects associated with ARS have been reported, but the extent of the cardiac defects has yet to be defined. We report a case of a 17-year-old girl with ARS with typical facial malformations and severe mitral and tricuspid valve insufficiency. The patient was diagnosed with secondary glaucoma detected on ophthalmologic examination. Echocardiography showed severe mitral and tricuspid valve insufficiency. This case provides further evidence of the association of ARS with cardiac malformations and extends the reported range of cardiac malformations in patients with ARS.

## Background

Axenfeld-Rieger anomaly (ARA) is an ocular condition characterized by extensive defects of the anterior chamber of the eye, mainly affecting the corneal structures. Anterior chamber defects include Axenfeld anomaly, characterized by prominent and anteriorly displaced Schwalbe lines and iris-corneal adhesions, and Rieger anomaly, which manifests as iris hypoplasia and pupil malformation (1). These ocular changes may lead to secondary glaucoma. Secondary glaucoma occurs due to ocular or systemic diseases that affect or destroy the normal aqueous humor circulation, block aqueous humor discharge, and cause intraocular pressure elevation. The presence of anomalies in the anterior chamber angle and drainage structures of the eye contributes to a lifetime risk of glaucoma and can lead to irreversible blindness (2). ARA can occur as an isolated defect, be episodic, an autosomal dominant trait, or part of a syndrome. ARA is sometimes associated with extraocular developmental defects, particularly of the teeth, facial bones, and periaqueductal skin (3). The combination of ocular and extraocular malformations is known as Axenfeld-Rieger syndrome (ARS) (4). The disorders that make up ARS are complex, inherited in a dominant manner, have a high ectopic rate, and are genetically heterogeneous (5). ARS is classified into three types based on systemic manifestations. Patients with ARS type 1 typically present with dental anomalies, craniofacial anomalies, and umbilical anomalies; patients with ARS type 2 usually present with oligodontia and microdontia, but craniofacial and umbilical anomalies are less common; patients with ARS type 3 rarely present with significant dental or facial anomalies, but may have hearing loss and heart defects (6–8). Herein, we report a rare case of a patient with ARS with both distinctive facial malformations and mitral and tricuspid valve dysplasia.

## Case presentation

**Figure 1:**
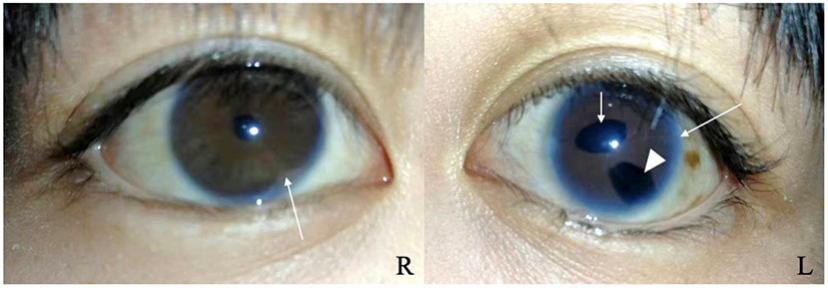
Slit-lamp photographs of the right and left eyes. Both eyes show anterior synechia (long arrows). The left eye shows pupil deformation and deviation (short arrow), iris atrophy causing the formation of a localized pore (arrowhead).

**Figure 2:**
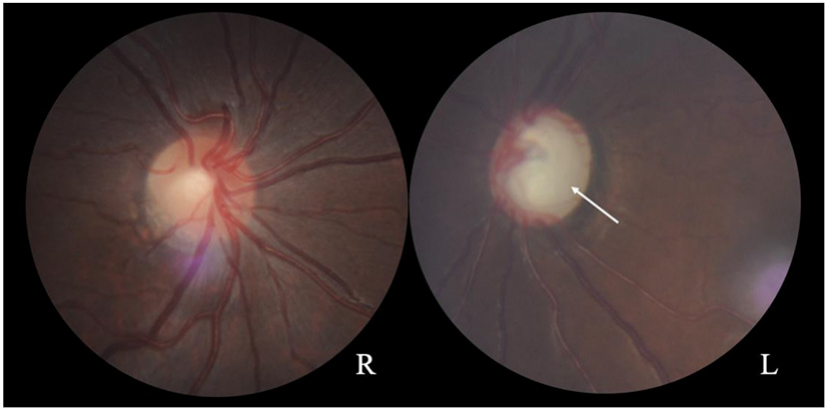
Fundus photographs showing a normal optic disc in the right eye and a pale optic disc with a clear border in the left eye (arrow). The cup-to-disc ratio is 0.3 for the right eye and 0.9 for the left eye.

**Figure 3:**
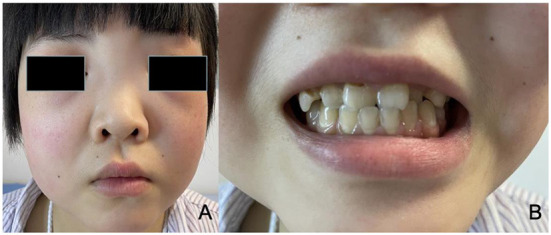
**(A)** The patient has a wide flat nasal bridge, increased distance between the medial canthi, and flat cheeks. **(B)** The patient has fairly normal dentition with a sharp crown and poorly developed permanent teeth.

A 17-year-old girl was admitted to the hospital 1 year ago for shortness of breath after activity, dyspnea, weakness, and generalized swelling with no apparent reason. The patient then visited us for investigation of bilateral secondary glaucoma. The patient had poor vision in her left eye and could only see the optometrist's hand moving at 30 cm from her face. Ocular examination revealed visual acuity of 0.5 in the right eye, intraocular pressure of 28.2 mmHg in the right eye and 39.8 mmHg in the left eye; the left eye had 15 degrees of exotropia, a corneal diameter of 13 mm, slight edema, partial anterior adhesion of the atrial angle, iris atrophy, and localized hole formation (Figure 1). She had a distorted and deviated left pupil, clear right cornea, round right pupil, clear lens in both eyes, cup-to-disc ratio of 0.9 in the left eye and 0.3 in the right eye, pale and well-defined optic disc in the left eye, and normal retinas (Figure 2). Atrial angle examination revealed extensive anterior adhesions of the iris in both eyes, anterior displacement of Schwalbe's ring, and striated tissue across the atrial angle in the peripheral part Bof the iris, adhering to Schwalbe's line and pulling on the iris. The midface was flattened and the eyes were widely spaced (Figure 3A). The dentition was still regular, with a sharp crown and poorly developed permanent teeth, of which there were currently only four (Figure 3B). The heart borders were enlarged on percussion. On auscultation, the heart rate was 87 beats/min without arrhythmia, with systolic murmurs (grade 3/6) in the mitral and tricuspid valve areas.

**Figure 4:**
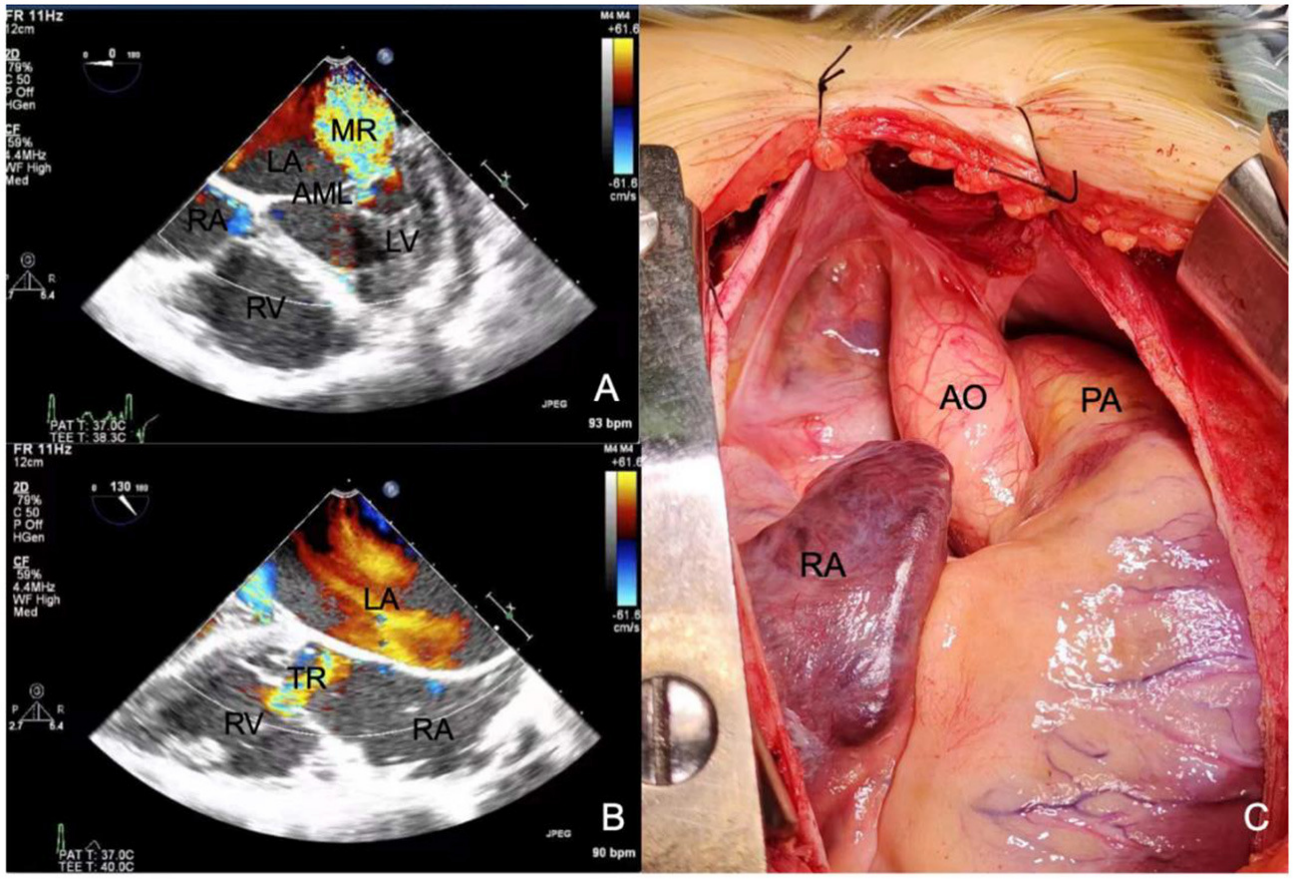
Echocardiography shows **(A)** severe mitral regurgitation and **(B)** severe tricuspid regurgitation. **(C)** Intraoperative photographs show that the aorta is 1.5 cm in diameter, which is significantly smaller than normal. RA, right atrium; RV, right ventricle; LA, left atrium; LV, left ventricle; AML, anterior mitral leaflet; MR, mitral regurgitation; TR, tricuspid regurgitation; AO, aorta; PA, pulmonary artery.

The patient was diagnosed with ARS and secondary glaucoma in both eyes, and anti-glaucoma surgery was recommended. Considering the patient's young age, advanced glaucoma, and the risk of non-compliance during awake surgery, anti-glaucoma surgery under general anesthesia was proposed to reduce the intraoperative stimulation of the optic nerve. Preoperative echocardiography showed a left ventricular ejection fraction of 58%, mitral valve thickening and severe mitral valve closure incompetence (Figure 4A), total heart enlargement, severe tricuspid valve closure incompetence (Figure 4B), and reduced right ventricular systolic function. These findings showed that the patient had poor cardiac function and was potentially unable to tolerate ocular surgery. Therefore, she was treated with diuretics to lower the intraocular pressure and underwent cardiac surgery to improve her cardiac function. The patient underwent mitral and tricuspid valvuloplasty with a #28 Sovering band in the mitral valve and a #27 Sovering band in the tricuspid valve. Intraoperatively, the patient was also found to have a hypoplastic aorta with a significantly smaller aortic diameter than normal for her age (Figure 4C). The patient recovered well postoperatively, and postoperative echocardiography showed normal valve function.

## Discussion

There are previous reports of ARS in association with congenital cardiac anomalies such as mitral, aortic, and pulmonary valve lesions, atrial septal defects, ventricular septal defects, and arterial trunk malformations (9–14). Gripp et al. (10) reported a 21-month-old patient with glaucoma who had congestive heart failure due to a dysplastic arched mitral valve and a mildly dysplastic left ventricular outflow tract and aortic valve. Grosso et al. (15) reported a patient with both mitral valve prolapse and tricuspid stenosis who presented with mitral and tricuspid valve insufficiency after two valvuloplasty procedures. Their patient's father underwent echocardiography for recurrent dyspnea, which was suggestive of mitral valve prolapse and tricuspid valve insufficiency (15). The family reported by Grosso et al. (15) is unusual in that they had no characteristic facial features of ARS, such as widely spaced eyes, a wide flat nasal bridge, flat cheeks, an acutely protruding lower jaw, an underbite, missing teeth, sparse teeth, a sharp crown, a central notch in the crown of the tooth, or degenerated periungual skin (16). Grosso et al. (15) concluded that the genetic characteristics of the family members were interrelated and not coincidental, and suggested that their findings might further support the hypothesis of a new genetic syndrome as proposed by Cunningham et al. (17). In the present case, we reported a 17-year-old girl with ARS with secondary glaucoma who had characteristic significant facial deformities and dental hypoplasia. Our patient also presented with severe mitral and tricuspid valve insufficiency, and had a small aortic diameter. Leaflet plication and repair were successful and well-tolerated intraoperatively.

Numerous studies have shown that the ocular and extra-ocular manifestations of ARS are associated with neural crest dysplasia. Besides, some researchers have suggested that neural crest dysplasia is related to the pathogenesis of aortic constriction and mitral aortic valve disease, as neural crest cells are also present in the heart valves (11, 18). Similarly, we believe that the heart valve defects observed in our patient were attributable to a developmental disorder of the neural crest tissue. To date, three loci associated with ARS have been identified on chromosomes 4q25, 6p25, and 13q14 (19–21). Genes for 4q25 and 6p25 have been cloned and are known as *PITX2* and *FOXC1*, respectively (3, 22, 23). *PITX2* is a pair of homologous frameshift genes that regulate the expression of other genes during embryonic development. Lu et al. (24). suggested that the *PITX2* product may be an effector from the early embryo stage to the formation of the left and right axes of each organ. Expression of the homologous frame gene *PITX2* in the neural crest is required for the development of the optic stalk and preoptic ganglion. Studies have demonstrated a direct link between *PITX2* deficiency and abnormal development of cardiac structures, including the atrioventricular valve (25, 26). Although *PITX2c* is a major transcript in mouse and human embryonic and adult hearts and is primarily responsible for cardiogenesis, *PITX2a* and *PITX2b* are expressed simultaneously during heart development and play an essential role in the development of the heart (27–29).

The presence of congenital heart malformations in ARS is reportedly associated with *FOXC1* mutations (30–32). Swiderski et al. (33) showed that the mouse homolog of *FOXC1, Mf1*, contributes not only to cardiac neural crest cell-derived structures but also to a range of embryonic tissue-derived structures, including the endocardial cushion cells that form the mitral and tricuspid valves; they also referenced cases with mitral and tricuspid valve insufficiency that support the possibility that the *FOXC1* gene may have an important effect on mitral and tricuspid valve insufficiency in ARS (33). The existing evidence suggests that individuals with the *FOXC1* variant are generally more likely to exhibit an isolated ocular phenotype or to exhibit a range of systemic features, of which heart defects, hearing loss, and growth delay are the most common. In contrast, the *PITX2* variant is closely associated with the ocular phenotype with dental anomalies (microdontia, hypodontia) and cord defects (periumbilical skin, umbilical hernia) (30, 31, 34, 35).

## Conclusion

The present patient had the distinct ocular phenotype, dental anomalies, and mitral and tricuspid valve manifestations of ARS. The findings in this patient expand the range of cardiac malformations reported in ARS patients. We believe that echocardiography should be performed in patients with characteristic clinical manifestations of ARS or with alterations in the *PITX2* and *FOXC1* genes.

## Data availability statement

The original contributions presented in the study are included in the article/supplementary material, further inquiries can be directed to the corresponding author.

## Author contributions

JF and YW wrote the manuscript. SC collected the patient information and data. ZL, LL, and QM participated in the formulation and management of the patient's perioperative treatment plan. CZ designed and reviewed the manuscript. All authors were involved in the revision of the manuscript and approved the submitted version.

## Conflict of interest

The authors declare that the research was conducted in the absence of any commercial or financial relationships that could be construed as a potential conflict of interest.

## Publisher's note

All claims expressed in this article are solely those of the authors and do not necessarily represent those of their affiliated organizations, or those of the publisher, the editors and the reviewers. Any product that may be evaluated in this article, or claim that may be made by its manufacturer, is not guaranteed or endorsed by the publisher.
